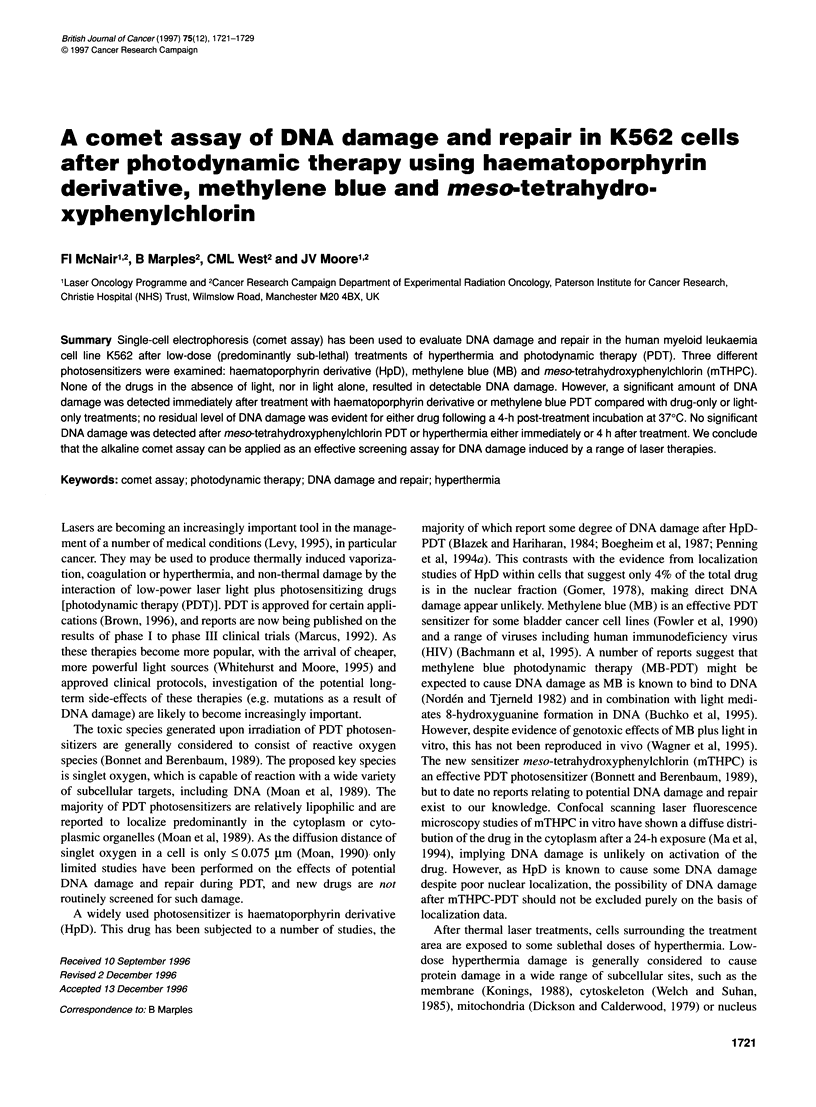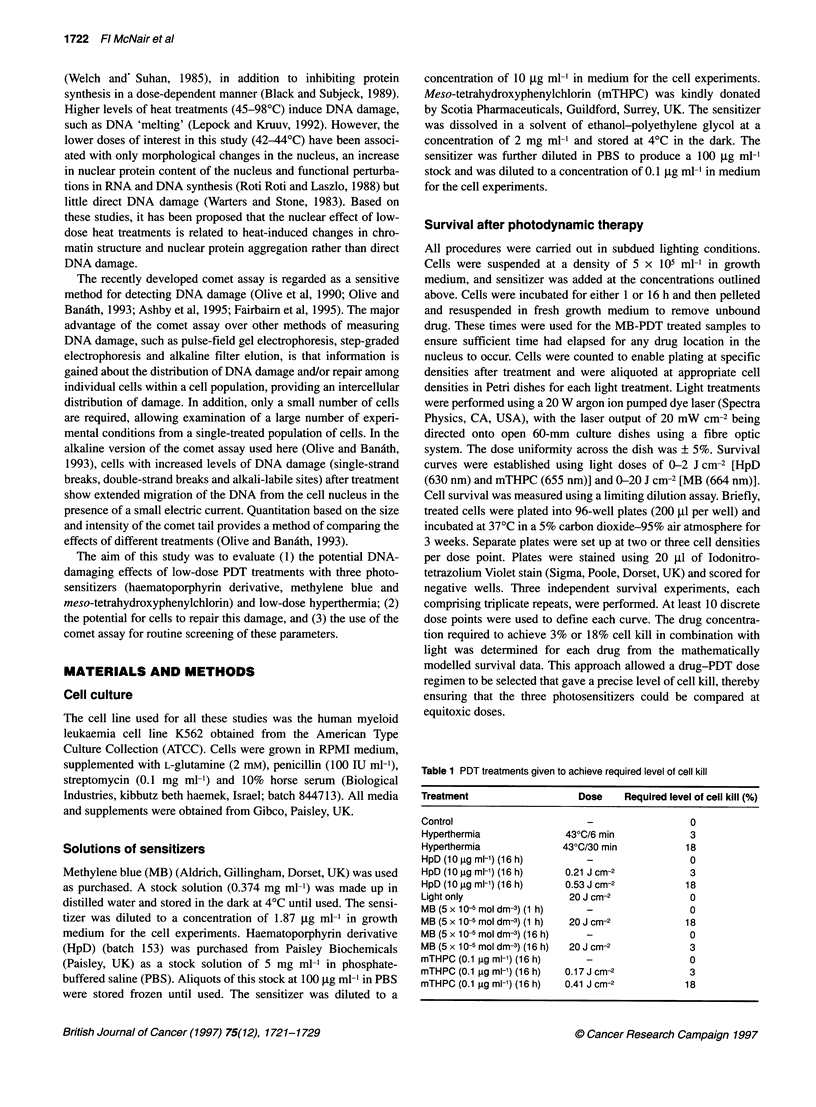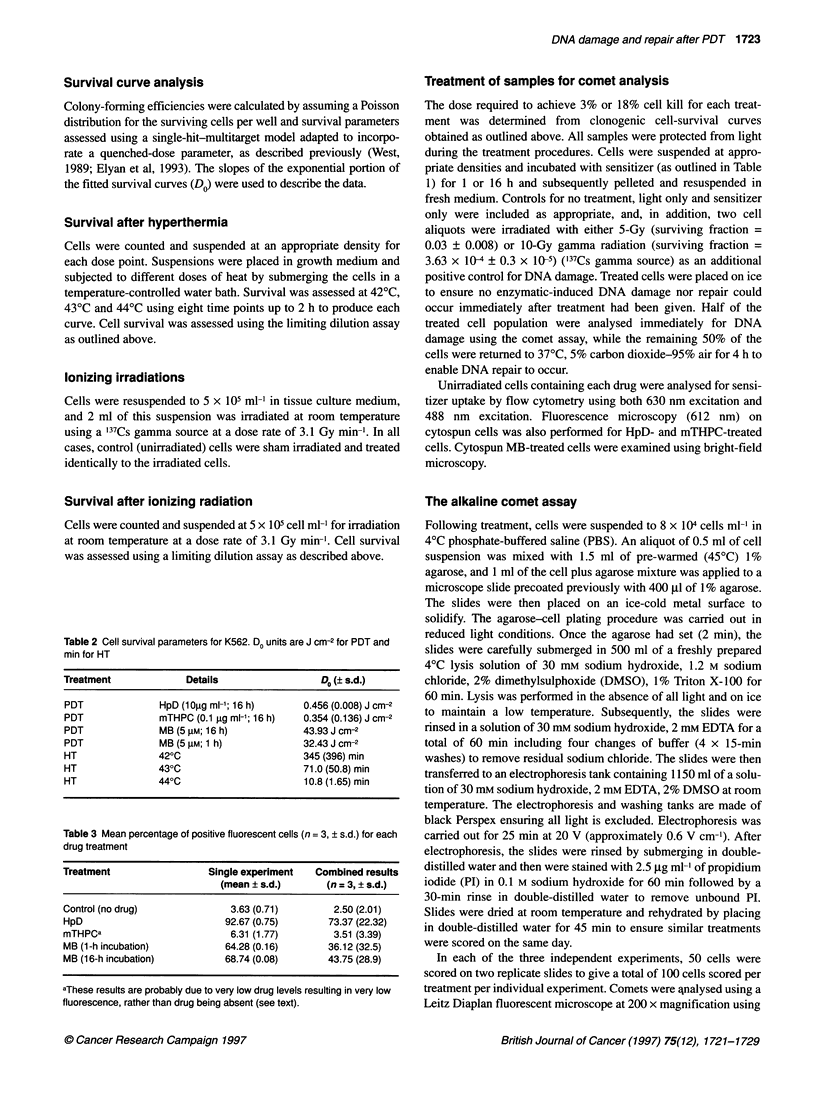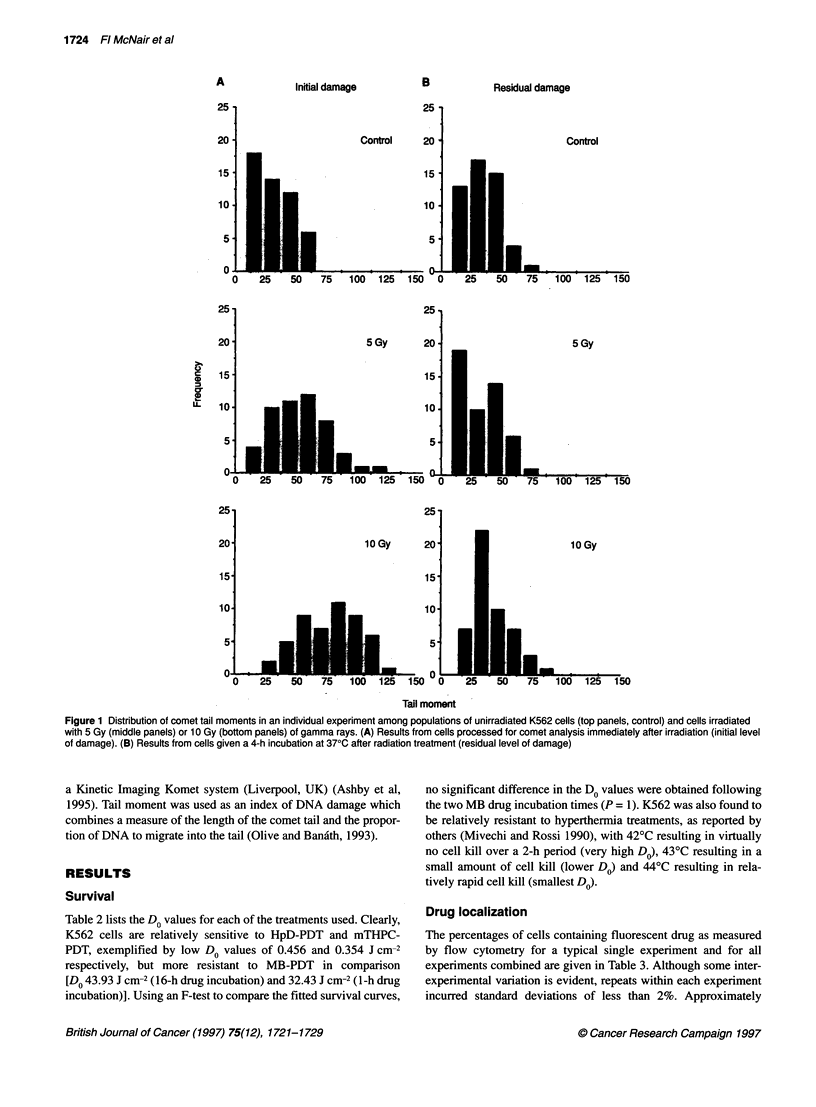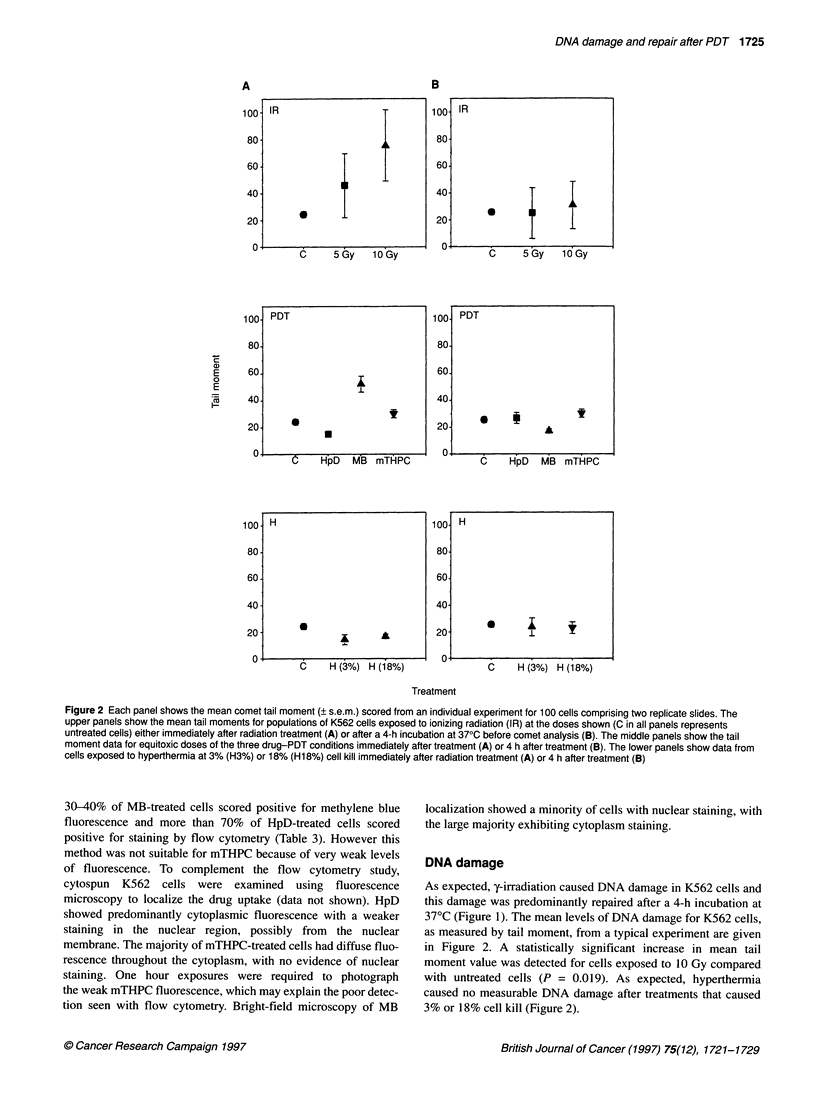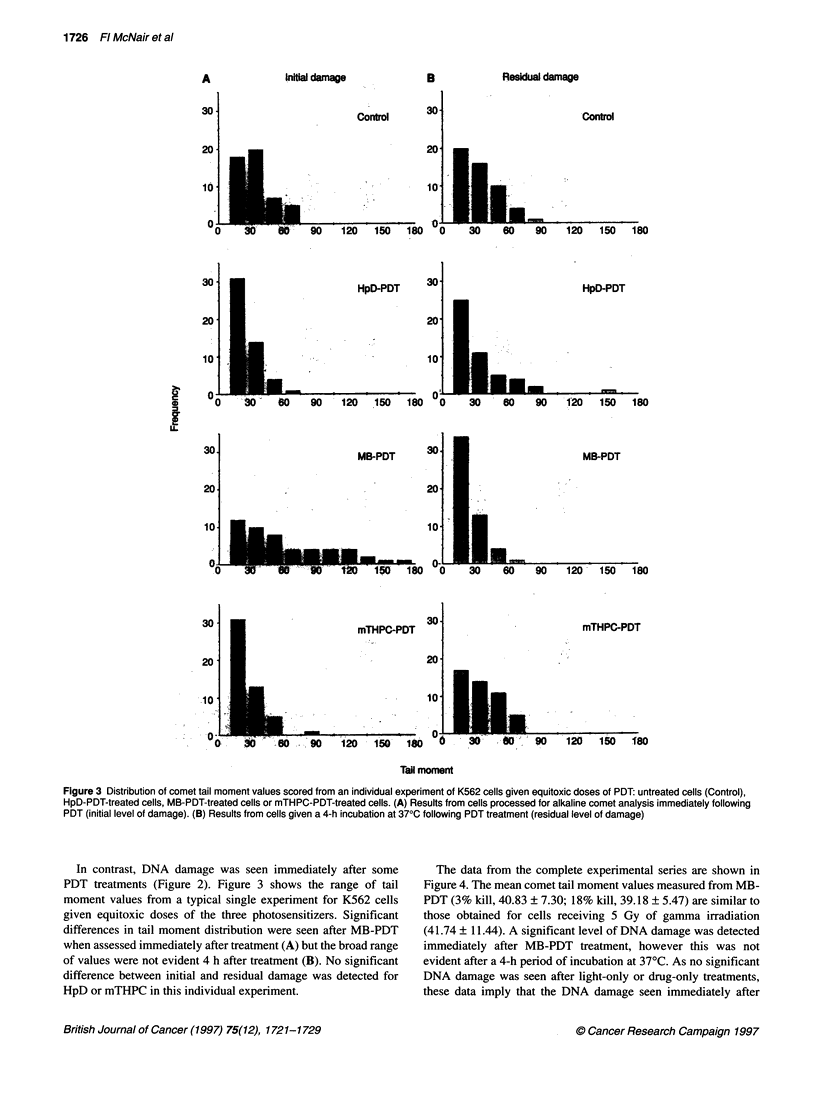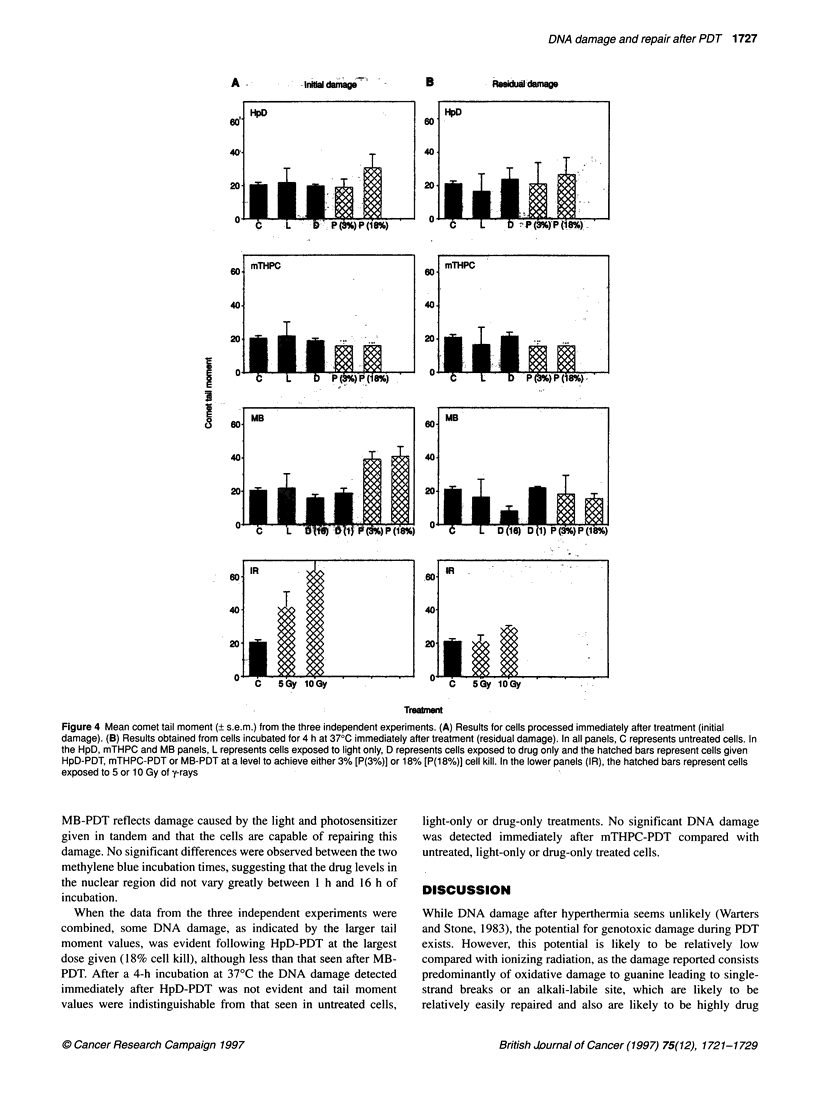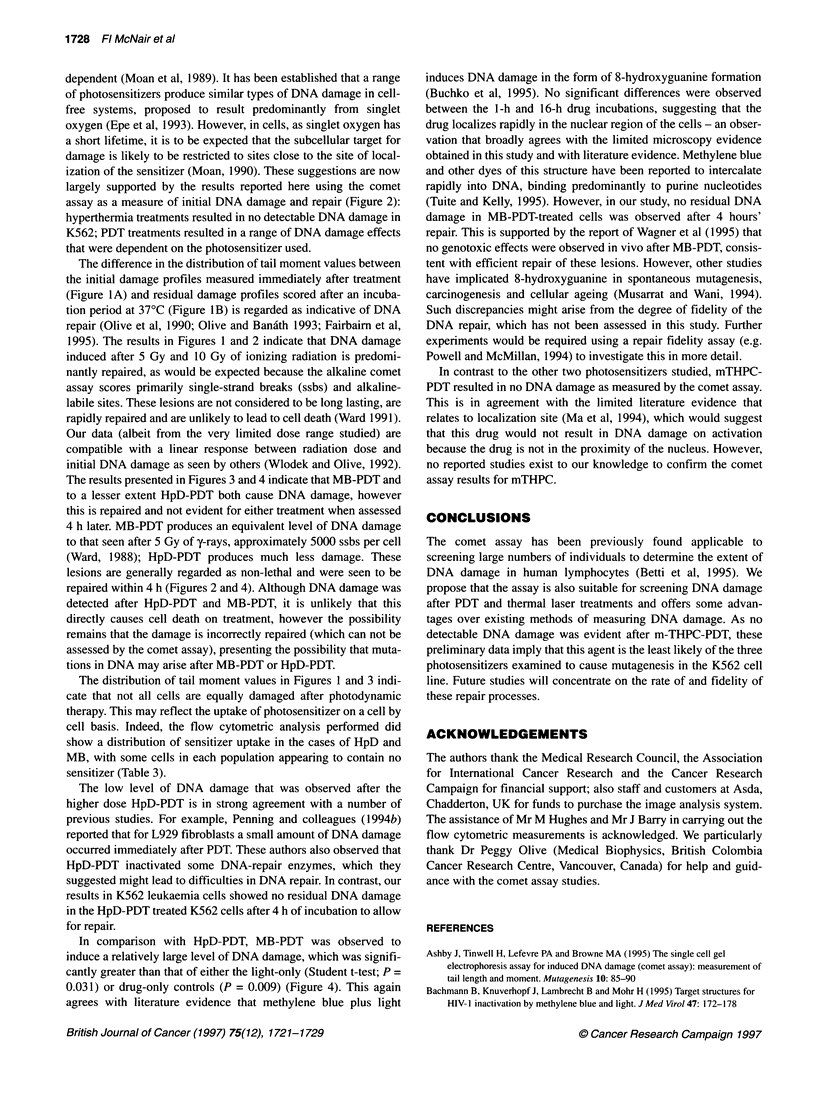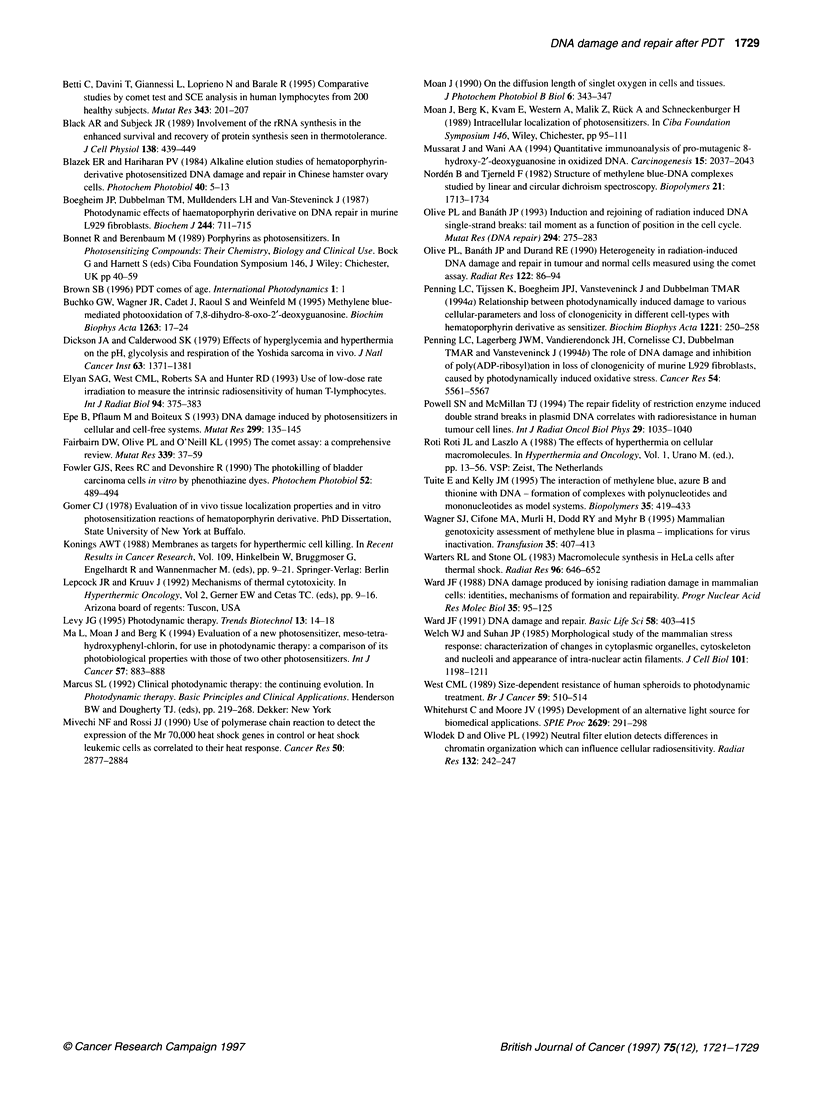# A comet assay of DNA damage and repair in K562 cells after photodynamic therapy using haematoporphyrin derivative, methylene blue and meso-tetrahydroxyphenylchlorin.

**DOI:** 10.1038/bjc.1997.295

**Published:** 1997

**Authors:** F. I. McNair, B. Marples, C. M. West, J. V. Moore

**Affiliations:** Laser Oncology Programme and Cancer Research Campaign Department of Experimental Radiation Oncology, Paterson Institute for Cancer Research, Christie Hospital (NHS) Trust, Manchester, UK.

## Abstract

Single-cell electrophoresis (comet assay) has been used to evaluate DNA damage and repair in the human myeloid leukaemia cell line K562 after low-dose (predominantly sub-lethal) treatments of hyperthermia and photodynamic therapy (PDT). Three different photosensitizers were examined: haematoporphyrin derivative (HpD), methylene blue (MB) and meso-tetrahydroxyphenylchlorin (mTHPC). None of the drugs in the absence of light, nor in light alone, resulted in detectable DNA damage. However, a significant amount of DNA damage was detected immediately after treatment with haematoporphyrin derivative or methylene blue PDT compared with drug-only or light-only treatments; no residual level of DNA damage was evident for either drug following a 4-h post-treatment incubation at 37 degrees C. No significant DNA damage was detected after meso-tetrahydroxyphenylchlorin PDT or hyperthermia either immediately or 4 h after treatment. We conclude that the alkaline comet assay can be applied as an effective screening assay for DNA damage induced by a range of laser therapies.


British Journal of Cancer (1997) 75(12), 1721–1729
© 1997 Cancer Research Campaign

### A comet assay of DNA damage and repair in K562 cells after photodynamic therapy using haematoporphyrin derivative, methylene blue and *meso*-tetrahydroxyphenylchlorin

FI McNair[1,2], B Marples[2], CML West[2] and JV Moore[1,2]

[1]Laser Oncology Programme and [2]Cancer Research Campaign Department of Experimental Radiation Oncology, Paterson Institute for Cancer Research, Christie Hospital (NHS) Trust, Wilmslow Road, Manchester M20 4BX, UK

**Summary** Single-cell electrophoresis (comet assay) has been used to evaluate DNA damage and repair in the human myeloid leukaemia cell line K562 after low-dose (predominantly sub-lethal) treatments of hyperthermia and photodynamic therapy (PDT). Three different photosensitizers were examined: haematoporphyrin derivative (HpD), methylene blue (MB) and *meso*-tetrahydroxyphenylchlorin (mTHPC). None of the drugs in the absence of light, nor in light alone, resulted in detectable DNA damage. However, a significant amount of DNA damage was detected immediately after treatment with haematoporphyrin derivative or methylene blue PDT compared with drug-only or light-only treatments; no residual level of DNA damage was evident for either drug following a 4-h post-treatment incubation at 37°C. No significant DNA damage was detected after *meso*-tetrahydroxyphenylchlorin PDT or hyperthermia either immediately or 4 h after treatment. We conclude that the alkaline comet assay can be applied as an effective screening assay for DNA damage induced by a range of laser therapies.

**Keywords:** comet assay; photodynamic therapy; DNA damage and repair; hyperthermia

Lasers are becoming an increasingly important tool in the management of a number of medical conditions (Levy, 1995), in particular cancer. They may be used to produce thermally induced vaporization, coagulation or hyperthermia, and non-thermal damage by the interaction of low-power laser light plus photosensitizing drugs [photodynamic therapy (PDT)]. PDT is approved for certain applications (Brown, 1996), and reports are now being published on the results of phase I to phase III clinical trials (Marcus, 1992). As these therapies become more popular, with the arrival of cheaper, more powerful light sources (Whitehurst and Moore, 1995) and approved clinical protocols, investigation of the potential long-term side-effects of these therapies (e.g. mutations as a result of DNA damage) are likely to become increasingly important.

The toxic species generated upon irradiation of PDT photosensitizers are generally considered to consist of reactive oxygen species (Bonnet and Berenbaum, 1989). The proposed key species is singlet oxygen, which is capable of reaction with a wide variety of subcellular targets, including DNA (Moan et al, 1989). The majority of PDT photosensitizers are relatively lipophilic and are reported to localize predominantly in the cytoplasm or cytoplasmic organelles (Moan et al, 1989). As the diffusion distance of singlet oxygen in a cell is only $\leq 0.075$ μm (Moan, 1990) only limited studies have been performed on the effects of potential DNA damage and repair during PDT, and new drugs are *not* routinely screened for such damage.

A widely used photosensitizer is haematoporphyrin derivative (HpD). This drug has been subjected to a number of studies, the majority of which report some degree of DNA damage after HpD-PDT (Blazek and Hariharan, 1984; Boegheim et al, 1987; Penning et al, 1994*a*). This contrasts with the evidence from localization studies of HpD within cells that suggest only 4% of the total drug is in the nuclear fraction (Gomer, 1978), making direct DNA damage appear unlikely. Methylene blue (MB) is an effective PDT sensitizer for some bladder cancer cell lines (Fowler et al, 1990) and a range of viruses including human immunodeficiency virus (HIV) (Bachmann et al, 1995). A number of reports suggest that methylene blue photodynamic therapy (MB-PDT) might be expected to cause DNA damage as MB is known to bind to DNA (Nordén and Tjerneld 1982) and in combination with light mediates 8-hydroxyguanine formation in DNA (Buchko et al, 1995). However, despite evidence of genotoxic effects of MB plus light in vitro, this has not been reproduced in vivo (Wagner et al, 1995). The new sensitizer *meso*-tetrahydroxyphenylchlorin (mTHPC) is an effective PDT photosensitizer (Bonnett and Berenbaum, 1989), but to date no reports relating to potential DNA damage and repair exist to our knowledge. Confocal scanning laser fluorescence microscopy studies of mTHPC in vitro have shown a diffuse distribution of the drug in the cytoplasm after a 24-h exposure (Ma et al, 1994), implying DNA damage is unlikely on activation of the drug. However, as HpD is known to cause some DNA damage despite poor nuclear localization, the possibility of DNA damage after mTHPC-PDT should not be excluded purely on the basis of localization data.

After thermal laser treatments, cells surrounding the treatment area are exposed to some sublethal doses of hyperthermia. Low-dose hyperthermia damage is generally considered to cause protein damage in a wide range of subcellular sites, such as the membrane (Konings, 1988), cytoskeleton (Welch and Suhan, 1985), mitochondria (Dickson and Calderwood, 1979) or nucleus

*Received 10 September 1996*
*Revised 2 December 1996*
*Accepted 13 December 1996*

*Correspondence to:* B Marples

(Welch and' Suhan, 1985), in addition to inhibiting protein synthesis in a dose-dependent manner (Black and Subjeck, 1989). Higher levels of heat treatments (45–98°C) induce DNA damage, such as DNA 'melting' (Lepock and Kruuv, 1992). However, the lower doses of interest in this study (42–44°C) have been associated with only morphological changes in the nucleus, an increase in nuclear protein content of the nucleus and functional perturbations in RNA and DNA synthesis (Roti Roti and Laszlo, 1988) but little direct DNA damage (Warters and Stone, 1983). Based on these studies, it has been proposed that the nuclear effect of low-dose heat treatments is related to heat-induced changes in chromatin structure and nuclear protein aggregation rather than direct DNA damage.

The recently developed comet assay is regarded as a sensitive method for detecting DNA damage (Olive et al, 1990; Olive and Banáth, 1993; Ashby et al, 1995; Fairbairn et al, 1995). The major advantage of the comet assay over other methods of measuring DNA damage, such as pulse-field gel electrophoresis, step-graded electrophoresis and alkaline filter elution, is that information is gained about the distribution of DNA damage and/or repair among individual cells within a cell population, providing an intercellular distribution of damage. In addition, only a small number of cells are required, allowing examination of a large number of experimental conditions from a single-treated population of cells. In the alkaline version of the comet assay used here (Olive and Banáth, 1993), cells with increased levels of DNA damage (single-strand breaks, double-strand breaks and alkali-labile sites) after treatment show extended migration of the DNA from the cell nucleus in the presence of a small electric current. Quantitation based on the size and intensity of the comet tail provides a method of comparing the effects of different treatments (Olive and Banáth, 1993).

The aim of this study was to evaluate (1) the potential DNA-damaging effects of low-dose PDT treatments with three photosensitizers (haematoporphyrin derivative, methylene blue and *meso*-tetrahydroxyphenylchlorin) and low-dose hyperthermia; (2) the potential for cells to repair this damage, and (3) the use of the comet assay for routine screening of these parameters.

### MATERIALS AND METHODS

### Cell culture

The cell line used for all these studies was the human myeloid leukaemia cell line K562 obtained from the American Type Culture Collection (ATCC). Cells were grown in RPMI medium, supplemented with L-glutamine (2 mM), penicillin (100 IU ml$^{-1}$), streptomycin (0.1 mg ml$^{-1}$) and 10% horse serum (Biological Industries, kibbutz beth haemek, Israel; batch 844713). All media and supplements were obtained from Gibco, Paisley, UK.

### Solutions of sensitizers

Methylene blue (MB) (Aldrich, Gillingham, Dorset, UK) was used as purchased. A stock solution (0.374 mg ml$^{-1}$) was made up in distilled water and stored in the dark at 4°C until used. The sensitizer was diluted to a concentration of 1.87 μg ml$^{-1}$ in growth medium for the cell experiments. Haematoporphyrin derivative (HpD) (batch 153) was purchased from Paisley Biochemicals (Paisley, UK) as a stock solution of 5 mg ml$^{-1}$ in phosphate-buffered saline (PBS). Aliquots of this stock at 100 μg ml$^{-1}$ in PBS were stored frozen until used. The sensitizer was diluted to a concentration of 10 μg ml$^{-1}$ in medium for the cell experiments. *Meso*-tetrahydroxyphenylchlorin (mTHPC) was kindly donated by Scotia Pharmaceuticals, Guildford, Surrey, UK. The sensitizer was dissolved in a solvent of ethanol–polyethylene glycol at a concentration of 2 mg ml$^{-1}$ and stored at 4°C in the dark. The sensitizer was further diluted in PBS to produce a 100 μg ml$^{-1}$ stock and was diluted to a concentration of 0.1 μg ml$^{-1}$ in medium for the cell experiments.

### Survival after photodynamic therapy

All procedures were carried out in subdued lighting conditions. Cells were suspended at a density of $5 \times 10^5$ ml$^{-1}$ in growth medium, and sensitizer was added at the concentrations outlined above. Cells were incubated for either 1 or 16 h and then pelleted and resuspended in fresh growth medium to remove unbound drug. These times were used for the MB-PDT treated samples to ensure sufficient time had elapsed for any drug location in the nucleus to occur. Cells were counted to enable plating at specific densities after treatment and were aliquoted at appropriate cell densities in Petri dishes for each light treatment. Light treatments were performed using a 20 W argon ion pumped dye laser (Spectra Physics, CA, USA), with the laser output of 20 mW cm$^{-2}$ being directed onto open 60-mm culture dishes using a fibre optic system. The dose uniformity across the dish was ± 5%. Survival curves were established using light doses of 0–2 J cm$^{-2}$ [HpD (630 nm) and mTHPC (655 nm)] and 0–20 J cm$^{-2}$ [MB (664 nm)]. Cell survival was measured using a limiting dilution assay. Briefly, treated cells were plated into 96-well plates (200 μl per well) and incubated at 37°C in a 5% carbon dioxide–95% air atmosphere for 3 weeks. Separate plates were set up at two or three cell densities per dose point. Plates were stained using 20 μl of Iodonitrotetrazolium Violet stain (Sigma, Poole, Dorset, UK) and scored for negative wells. Three independent survival experiments, each comprising triplicate repeats, were performed. At least 10 discrete dose points were used to define each curve. The drug concentration required to achieve 3% or 18% cell kill in combination with light was determined for each drug from the mathematically modelled survival data. This approach allowed a drug–PDT dose regimen to be selected that gave a precise level of cell kill, thereby ensuring that the three photosensitizers could be compared at equitoxic doses.

**Table 1** PDT treatments given to achieve required level of cell kill

| Treatment | Dose | Required level of cell kill (%) |
|---|---|---|
| Control | – | 0 |
| Hyperthermia | 43°C/6 min | 3 |
| Hyperthermia | 43°C/30 min | 18 |
| HpD (10 μg ml$^{-1}$) (16 h) | – | 0 |
| HpD (10 μg ml$^{-1}$) (16 h) | 0.21 J cm$^{-2}$ | 3 |
| HpD (10 μg ml$^{-1}$) (16 h) | 0.53 J cm$^{-2}$ | 18 |
| Light only | 20 J cm$^{-2}$ | 0 |
| MB ($5 \times 10^{-5}$ mol dm$^{-3}$) (1 h) | – | 0 |
| MB ($5 \times 10^{-5}$ mol dm$^{-3}$) (1 h) | 20 J cm$^{-2}$ | 18 |
| MB ($5 \times 10^{-5}$ mol dm$^{-3}$) (16 h) | – | 0 |
| MB ($5 \times 10^{-5}$ mol dm$^{-3}$) (16 h) | 20 J cm$^{-2}$ | 3 |
| mTHPC (0.1 μg ml$^{-1}$) (16 h) | – | 0 |
| mTHPC (0.1 μg ml$^{-1}$) (16 h) | 0.17 J cm$^{-2}$ | 3 |
| mTHPC (0.1 μg ml$^{-1}$) (16 h) | 0.41 J cm$^{-2}$ | 18 |

© Cancer Research Campaign 1997

### Survival curve analysis

Colony-forming efficiencies were calculated by assuming a Poisson distribution for the surviving cells per well and survival parameters assessed using a single-hit–multitarget model adapted to incorporate a quenched-dose parameter, as described previously (West, 1989; Elyan et al, 1993). The slopes of the exponential portion of the fitted survival curves ($D_0$) were used to describe the data.

### Survival after hyperthermia

Cells were counted and suspended at an appropriate density for each dose point. Suspensions were placed in growth medium and subjected to different doses of heat by submerging the cells in a temperature-controlled water bath. Survival was assessed at 42°C, 43°C and 44°C using eight time points up to 2 h to produce each curve. Cell survival was assessed using the limiting dilution assay as outlined above.

### Ionizing irradiations

Cells were resuspended to $5 \times 10^5$ ml$^{-1}$ in tissue culture medium, and 2 ml of this suspension was irradiated at room temperature using a $^{137}$Cs gamma source at a dose rate of 3.1 Gy min$^{-1}$. In all cases, control (unirradiated) cells were sham irradiated and treated identically to the irradiated cells.

### Survival after ionizing radiation

Cells were counted and suspended at $5 \times 10^5$ cell ml$^{-1}$ for irradiation at room temperature at a dose rate of 3.1 Gy min$^{-1}$. Cell survival was assessed using a limiting dilution assay as described above.

**Table 2** Cell survival parameters for K562. $D_0$ units are J cm$^{-2}$ for PDT and min for HT

| Treatment | Details | $D_0$ (± s.d.) |
|---|---|---|
| PDT | HpD (10µg ml$^{-1}$; 16 h) | 0.456 (0.008) J cm$^{-2}$ |
| PDT | mTHPC (0.1 µg ml$^{-1}$; 16 h) | 0.354 (0.136) J cm$^{-2}$ |
| PDT | MB (5 µM; 16 h) | 43.93 J cm$^{-2}$ |
| PDT | MB (5 µM; 1 h) | 32.43 J cm$^{-2}$ |
| HT | 42°C | 345 (396) min |
| HT | 43°C | 71.0 (50.8) min |
| HT | 44°C | 10.8 (1.65) min |

**Table 3** Mean percentage of positive fluorescent cells ($n$ = 3, ± s.d.) for each drug treatment

| Treatment | Single experiment (mean ± s.d.) | Combined results ($n$ = 3, ± s.d.) |
|---|---|---|
| Control (no drug) | 3.63 (0.71) | 2.50 (2.01) |
| HpD | 92.67 (0.75) | 73.37 (22.32) |
| mTHPC[a] | 6.31 (1.77) | 3.51 (3.39) |
| MB (1-h incubation) | 64.28 (0.16) | 36.12 (32.5) |
| MB (16-h incubation) | 68.74 (0.08) | 43.75 (28.9) |

[a]These results are probably due to very low drug levels resulting in very low fluorescence, rather than drug being absent (see text).

### Treatment of samples for comet analysis

The dose required to achieve 3% or 18% cell kill for each treatment was determined from clonogenic cell-survival curves obtained as outlined above. All samples were protected from light during the treatment procedures. Cells were suspended at appropriate densities and incubated with sensitizer (as outlined in Table 1) for 1 or 16 h and subsequently pelleted and resuspended in fresh medium. Controls for no treatment, light only and sensitizer only were included as appropriate, and, in addition, two cell aliquots were irradiated with either 5-Gy (surviving fraction = 0.03 ± 0.008) or 10-Gy gamma radiation (surviving fraction = $3.63 \times 10^{-4} \pm 0.3 \times 10^{-5}$) ($^{137}$Cs gamma source) as an additional positive control for DNA damage. Treated cells were placed on ice to ensure no enzymatic-induced DNA damage nor repair could occur immediately after treatment had been given. Half of the treated cell population were analysed immediately for DNA damage using the comet assay, while the remaining 50% of the cells were returned to 37°C, 5% carbon dioxide–95% air for 4 h to enable DNA repair to occur.

Unirradiated cells containing each drug were analysed for sensitizer uptake by flow cytometry using both 630 nm excitation and 488 nm excitation. Fluorescence microscopy (612 nm) on cytospun cells was also performed for HpD- and mTHPC-treated cells. Cytospun MB-treated cells were examined using bright-field microscopy.

### The alkaline comet assay

Following treatment, cells were suspended to $8 \times 10^4$ cells ml$^{-1}$ in 4°C phosphate-buffered saline (PBS). An aliquot of 0.5 ml of cell suspension was mixed with 1.5 ml of pre-warmed (45°C) 1% agarose, and 1 ml of the cell plus agarose mixture was applied to a microscope slide precoated previously with 400 µl of 1% agarose. The slides were then placed on an ice-cold metal surface to solidify. The agarose–cell plating procedure was carried out in reduced light conditions. Once the agarose had set (2 min), the slides were carefully submerged in 500 ml of a freshly prepared 4°C lysis solution of 30 mM sodium hydroxide, 1.2 M sodium chloride, 2% dimethylsulphoxide (DMSO), 1% Triton X-100 for 60 min. Lysis was performed in the absence of all light and on ice to maintain a low temperature. Subsequently, the slides were rinsed in a solution of 30 mM sodium hydroxide, 2 mM EDTA for a total of 60 min including four changes of buffer (4 × 15-min washes) to remove residual sodium chloride. The slides were then transferred to an electrophoresis tank containing 1150 ml of a solution of 30 mM sodium hydroxide, 2 mM EDTA, 2% DMSO at room temperature. The electrophoresis and washing tanks are made of black Perspex ensuring all light is excluded. Electrophoresis was carried out for 25 min at 20 V (approximately 0.6 V cm$^{-1}$). After electrophoresis, the slides were rinsed by submerging in double-distilled water and then were stained with 2.5 µg ml$^{-1}$ of propidium iodide (PI) in 0.1 M sodium hydroxide for 60 min followed by a 30-min rinse in double-distilled water to remove unbound PI. Slides were dried at room temperature and rehydrated by placing in double-distilled water for 45 min to ensure similar treatments were scored on the same day.

In each of the three independent experiments, 50 cells were scored on two replicate slides to give a total of 100 cells scored per treatment per individual experiment. Comets were analysed using a Leitz Diaplan fluorescent microscope at 200 × magnification using

© Cancer Research Campaign 1997

**Figure 1** Distribution of comet tail moments in an individual experiment among populations of unirradiated K562 cells (top panels, control) and cells irradiated with 5 Gy (middle panels) or 10 Gy (bottom panels) of gamma rays. (**A**) Results from cells processed for comet analysis immediately after irradiation (initial level of damage). (**B**) Results from cells given a 4-h incubation at 37°C after radiation treatment (residual level of damage)

a Kinetic Imaging Komet system (Liverpool, UK) (Ashby et al, 1995). Tail moment was used as an index of DNA damage which combines a measure of the length of the comet tail and the proportion of DNA to migrate into the tail (Olive and Banáth, 1993).

### RESULTS

### Survival

Table 2 lists the $D_0$ values for each of the treatments used. Clearly, K562 cells are relatively sensitive to HpD-PDT and mTHPC-PDT, exemplified by low $D_0$ values of 0.456 and 0.354 J $cm^{-2}$ respectively, but more resistant to MB-PDT in comparison [$D_0$ 43.93 J $cm^{-2}$ (16-h drug incubation) and 32.43 J $cm^{-2}$ (1-h drug incubation)]. Using an F-test to compare the fitted survival curves, no significant difference in the $D_0$ values were obtained following the two MB drug incubation times ($P = 1$). K562 was also found to be relatively resistant to hyperthermia treatments, as reported by others (Mivechi and Rossi 1990), with 42°C resulting in virtually no cell kill over a 2-h period (very high $D_0$), 43°C resulting in a small amount of cell kill (lower $D_0$) and 44°C resulting in relatively rapid cell kill (smallest $D_0$).

### Drug localization

The percentages of cells containing fluorescent drug as measured by flow cytometry for a typical single experiment and for all experiments combined are given in Table 3. Although some inter-experimental variation is evident, repeats within each experiment incurred standard deviations of less than 2%. Approximately

© Cancer Research Campaign 1997

**Figure 2** Each panel shows the mean comet tail moment (± s.e.m.) scored from an individual experiment for 100 cells comprising two replicate slides. The upper panels show the mean tail moments for populations of K562 cells exposed to ionizing radiation (IR) at the doses shown (C in all panels represents untreated cells) either immediately after radiation treatment (**A**) or after a 4-h incubation at 37°C before comet analysis (**B**). The middle panels show the tail moment data for equitoxic doses of the three drug–PDT conditions immediately after treatment (**A**) or 4 h after treatment (**B**). The lower panels show data from cells exposed to hyperthermia at 3% (H3%) or 18% (H18%) cell kill immediately after radiation treatment (**A**) or 4 h after treatment (**B**)

30–40% of MB-treated cells scored positive for methylene blue fluorescence and more than 70% of HpD-treated cells scored positive for staining by flow cytometry (Table 3). However this method was not suitable for mTHPC because of very weak levels of fluorescence. To complement the flow cytometry study, cytospun K562 cells were examined using fluorescence microscopy to localize the drug uptake (data not shown). HpD showed predominantly cytoplasmic fluorescence with a weaker staining in the nuclear region, possibly from the nuclear membrane. The majority of mTHPC-treated cells had diffuse fluorescence throughout the cytoplasm, with no evidence of nuclear staining. One hour exposures were required to photograph the weak mTHPC fluorescence, which may explain the poor detection seen with flow cytometry. Bright-field microscopy of MB localization showed a minority of cells with nuclear staining, with the large majority exhibiting cytoplasm staining.

### DNA damage

As expected, γ-irradiation caused DNA damage in K562 cells and this damage was predominantly repaired after a 4-h incubation at 37°C (Figure 1). The mean levels of DNA damage for K562 cells, as measured by tail moment, from a typical experiment are given in Figure 2. A statistically significant increase in mean tail moment value was detected for cells exposed to 10 Gy compared with untreated cells ($P = 0.019$). As expected, hyperthermia caused no measurable DNA damage after treatments that caused 3% or 18% cell kill (Figure 2).

© Cancer Research Campaign 1997 

**Figure 3** Distribution of comet tail moment values scored from an individual experiment of K562 cells given equitoxic doses of PDT: untreated cells (Control), HpD-PDT-treated cells, MB-PDT-treated cells or mTHPC-PDT-treated cells. (**A**) Results from cells processed for alkaline comet analysis immediately following PDT (initial level of damage). (**B**) Results from cells given a 4-h incubation at 37°C following PDT treatment (residual level of damage)

In contrast, DNA damage was seen immediately after some PDT treatments (Figure 2). Figure 3 shows the range of tail moment values from a typical single experiment for K562 cells given equitoxic doses of the three photosensitizers. Significant differences in tail moment distribution were seen after MB-PDT when assessed immediately after treatment (**A**) but the broad range of values were not evident 4 h after treatment (**B**). No significant difference between initial and residual damage was detected for HpD or mTHPC in this individual experiment.

The data from the complete experimental series are shown in Figure 4. The mean comet tail moment values measured from MB-PDT (3% kill, 40.83 ± 7.30; 18% kill, 39.18 ± 5.47) are similar to those obtained for cells receiving 5 Gy of gamma irradiation (41.74 ± 11.44). A significant level of DNA damage was detected immediately after MB-PDT treatment, however this was not evident after a 4-h period of incubation at 37°C. As no significant DNA damage was seen after light-only or drug-only treatments, these data imply that the DNA damage seen immediately after

© Cancer Research Campaign 1997

**Figure 4** Mean comet tail moment (± s.e.m.) from the three independent experiments. (**A**) Results for cells processed immediately after treatment (initial damage). (**B**) Results obtained from cells incubated for 4 h at 37°C immediately after treatment (residual damage). In all panels, C represents untreated cells. In the HpD, mTHPC and MB panels, L represents cells exposed to light only, D represents cells exposed to drug only and the hatched bars represent cells given HpD-PDT, mTHPC-PDT or MB-PDT at a level to achieve either 3% [P(3%)] or 18% [P(18%)] cell kill. In the lower panels (IR), the hatched bars represent cells exposed to 5 or 10 Gy of γ-rays

MB-PDT reflects damage caused by the light and photosensitizer given in tandem and that the cells are capable of repairing this damage. No significant differences were observed between the two methylene blue incubation times, suggesting that the drug levels in the nuclear region did not vary greatly between 1 h and 16 h of incubation.

When the data from the three independent experiments were combined, some DNA damage, as indicated by the larger tail moment values, was evident following HpD-PDT at the largest dose given (18% cell kill), although less than that seen after MB-PDT. After a 4-h incubation at 37°C the DNA damage detected immediately after HpD-PDT was not evident and tail moment values were indistinguishable from that seen in untreated cells, light-only or drug-only treatments. No significant DNA damage was detected immediately after mTHPC-PDT compared with untreated, light-only or drug-only treated cells.

### DISCUSSION

While DNA damage after hyperthermia seems unlikely (Warters and Stone, 1983), the potential for genotoxic damage during PDT exists. However, this potential is likely to be relatively low compared with ionizing radiation, as the damage reported consists predominantly of oxidative damage to guanine leading to single-strand breaks or an alkali-labile site, which are likely to be relatively easily repaired and also are likely to be highly drug dependent (Moan et al, 1989). It has been established that a range of photosensitizers produce similar types of DNA damage in cell-free systems, proposed to result predominantly from singlet oxygen (Epe et al, 1993). However, in cells, as singlet oxygen has a short lifetime, it is to be expected that the subcellular target for damage is likely to be restricted to sites close to the site of localization of the sensitizer (Moan, 1990). These suggestions are now largely supported by the results reported here using the comet assay as a measure of initial DNA damage and repair (Figure 2): hyperthermia treatments resulted in no detectable DNA damage in K562; PDT treatments resulted in a range of DNA damage effects that were dependent on the photosensitizer used.

The difference in the distribution of tail moment values between the initial damage profiles measured immediately after treatment (Figure 1A) and residual damage profiles scored after an incubation period at 37°C (Figure 1B) is regarded as indicative of DNA repair (Olive et al, 1990; Olive and Banáth 1993; Fairbairn et al, 1995). The results in Figures 1 and 2 indicate that DNA damage induced after 5 Gy and 10 Gy of ionizing radiation is predominantly repaired, as would be expected because the alkaline comet assay scores primarily single-strand breaks (ssbs) and alkaline-labile sites. These lesions are not considered to be long lasting, are rapidly repaired and are unlikely to lead to cell death (Ward 1991). Our data (albeit from the very limited dose range studied) are compatible with a linear response between radiation dose and initial DNA damage as seen by others (Wlodek and Olive, 1992). The results presented in Figures 3 and 4 indicate that MB-PDT and to a lesser extent HpD-PDT both cause DNA damage, however this is repaired and not evident for either treatment when assessed 4 h later. MB-PDT produces an equivalent level of DNA damage to that seen after 5 Gy of $\gamma$-rays, approximately 5000 ssbs per cell (Ward, 1988); HpD-PDT produces much less damage. These lesions are generally regarded as non-lethal and were seen to be repaired within 4 h (Figures 2 and 4). Although DNA damage was detected after HpD-PDT and MB-PDT, it is unlikely that this directly causes cell death on treatment, however the possibility remains that the damage is incorrectly repaired (which can not be assessed by the comet assay), presenting the possibility that mutations in DNA may arise after MB-PDT or HpD-PDT.

The distribution of tail moment values in Figures 1 and 3 indicate that not all cells are equally damaged after photodynamic therapy. This may reflect the uptake of photosensitizer on a cell by cell basis. Indeed, the flow cytometric analysis performed did show a distribution of sensitizer uptake in the cases of HpD and MB, with some cells in each population appearing to contain no sensitizer (Table 3).

The low level of DNA damage that was observed after the higher dose HpD-PDT is in strong agreement with a number of previous studies. For example, Penning and colleagues (1994*b*) reported that for L929 fibroblasts a small amount of DNA damage occurred immediately after PDT. These authors also observed that HpD-PDT inactivated some DNA-repair enzymes, which they suggested might lead to difficulties in DNA repair. In contrast, our results in K562 leukaemia cells showed no residual DNA damage in the HpD-PDT treated K562 cells after 4 h of incubation to allow for repair.

In comparison with HpD-PDT, MB-PDT was observed to induce a relatively large level of DNA damage, which was significantly greater than that of either the light-only (Student t-test; $P$ = 0.031) or drug-only controls ($P$ = 0.009) (Figure 4). This again agrees with literature evidence that methylene blue plus light induces DNA damage in the form of 8-hydroxyguanine formation (Buchko et al, 1995). No significant differences were observed between the 1-h and 16-h drug incubations, suggesting that the drug localizes rapidly in the nuclear region of the cells – an observation that broadly agrees with the limited microscopy evidence obtained in this study and with literature evidence. Methylene blue and other dyes of this structure have been reported to intercalate rapidly into DNA, binding predominantly to purine nucleotides (Tuite and Kelly, 1995). However, in our study, no residual DNA damage in MB-PDT-treated cells was observed after 4 hours' repair. This is supported by the report of Wagner et al (1995) that no genotoxic effects were observed in vivo after MB-PDT, consistent with efficient repair of these lesions. However, other studies have implicated 8-hydroxyguanine in spontaneous mutagenesis, carcinogenesis and cellular ageing (Musarrat and Wani, 1994). Such discrepancies might arise from the degree of fidelity of the DNA repair, which has not been assessed in this study. Further experiments would be required using a repair fidelity assay (e.g. Powell and McMillan, 1994) to investigate this in more detail.

In contrast to the other two photosensitizers studied, mTHPC-PDT resulted in no DNA damage as measured by the comet assay. This is in agreement with the limited literature evidence that relates to localization site (Ma et al, 1994), which would suggest that this drug would not result in DNA damage on activation because the drug is not in the proximity of the nucleus. However, no reported studies exist to our knowledge to confirm the comet assay results for mTHPC.

### CONCLUSIONS

The comet assay has been previously found applicable to screening large numbers of individuals to determine the extent of DNA damage in human lymphocytes (Betti et al, 1995). We propose that the assay is also suitable for screening DNA damage after PDT and thermal laser treatments and offers some advantages over existing methods of measuring DNA damage. As no detectable DNA damage was evident after m-THPC-PDT, these preliminary data imply that this agent is the least likely of the three photosensitizers examined to cause mutagenesis in the K562 cell line. Future studies will concentrate on the rate of and fidelity of these repair processes.

### ACKNOWLEDGEMENTS

The authors thank the Medical Research Council, the Association for International Cancer Research and the Cancer Research Campaign for financial support; also staff and customers at Asda, Chadderton, UK for funds to purchase the image analysis system. The assistance of Mr M Hughes and Mr J Barry in carrying out the flow cytometric measurements is acknowledged. We particularly thank Dr Peggy Olive (Medical Biophysics, British Colombia Cancer Research Centre, Vancouver, Canada) for help and guidance with the comet assay studies.

### REFERENCES

Ashby J, Tinwell H, Lefevre PA and Browne MA (1995) The single cell gel electrophoresis assay for induced DNA damage (comet assay): measurement of tail length and moment. *Mutagenesis* **10**: 85–90

Bachmann B, Knuverhopf J, Lambrecht B and Mohr H (1995) Target structures for HIV-1 inactivation by methylene blue and light. *J Med Virol* **47**: 172–178

© Cancer Research Campaign 1997

Betti C, Davini T, Giannessi L, Loprieno N and Barale R (1995) Comparative studies by comet test and SCE analysis in human lymphocytes from 200 healthy subjects. *Mutat Res* **343**: 201–207

Black AR and Subjeck JR (1989) Involvement of the rRNA synthesis in the enhanced survival and recovery of protein synthesis seen in thermotolerance. *J Cell Physiol* **138**: 439–449

Blazek ER and Hariharan PV (1984) Alkaline elution studies of hematoporphyrin-derivative photosensitized DNA damage and repair in Chinese hamster ovary cells. *Photochem Photobiol* **40**: 5–13

Boegheim JP, Dubbelman TM, Mulldenders LH and Van-Steveninck J (1987) Photodynamic effects of haematoporphyrin derivative on DNA repair in murine L929 fibroblasts. *Biochem J* **244**: 711–715

Bonnet R and Berenbaum M (1989) Porphyrins as photosensitizers. In *Photosensitizing Compounds: Their Chemistry, Biology and Clinical Use*. Bock G and Harnett S (eds) Ciba Foundation Symposium 146, J Wiley: Chichester, UK pp 40–59

Brown SB (1996) PDT comes of age. *International Photodynamics* **1**: 1

Buchko GW, Wagner JR, Cadet J, Raoul S and Weinfeld M (1995) Methylene blue-mediated photooxidation of 7,8-dihydro-8-oxo-2′-deoxyguanosine. *Biochim Biophys Acta* **1263**: 17–24

Dickson JA and Calderwood SK (1979) Effects of hyperglycemia and hyperthermia on the pH, glycolysis and respiration of the Yoshida sarcoma in vivo. *J Natl Cancer Inst* **63**: 1371–1381

Elyan SAG, West CML, Roberts SA and Hunter RD (1993) Use of low-dose rate irradiation to measure the intrinsic radiosensitivity of human T-lymphocytes. *Int J Radiat Biol* **94**: 375–383

Epe B, Pflaum M and Boiteux S (1993) DNA damage induced by photosensitizers in cellular and cell-free systems. *Mutat Res* **299**: 135–145

Fairbairn DW, Olive PL and O'Neill KL (1995) The comet assay: a comprehensive review. *Mutat Res* **339**: 37–59

Fowler GJS, Rees RC and Devonshire R (1990) The photokilling of bladder carcinoma cells *in vitro* by phenothiazine dyes. *Photochem Photobiol* **52**: 489–494

Gomer CJ (1978) Evaluation of in vivo tissue localization properties and in vitro photosensitization reactions of hematoporphyrin derivative. PhD Dissertation, State University of New York at Buffalo.

Konings AWT (1988) Membranes as targets for hyperthermic cell killing. In *Recent Results in Cancer Research*, Vol. 109, Hinkelbein W, Bruggmoser G, Engelhardt R and Wannenmacher M. (eds), pp. 9–21. Springer-Verlag: Berlin

Lepcock JR and Kruuv J (1992) Mechanisms of thermal cytotoxicity. In *Hyperthermic Oncology*, Vol 2, Gerner EW and Cetas TC. (eds), pp. 9–16. Arizona board of regents: Tuscon, USA

Levy JG (1995) Photodynamic therapy. *Trends Biotechnol* **13**: 14–18

Ma L, Moan J and Berg K (1994) Evaluation of a new photosensitizer, meso-tetra-hydroxyphenyl-chlorin, for use in photodynamic therapy: a comparison of its photobiological properties with those of two other photosensitizers. *Int J Cancer* **57**: 883–888

Marcus SL (1992) Clinical photodynamic therapy: the continuing evolution. In *Photodynamic therapy. Basic Principles and Clinical Applications*. Henderson BW and Dougherty TJ. (eds), pp. 219–268. Dekker: New York

Mivechi NF and Rossi JJ (1990) Use of polymerase chain reaction to detect the expression of the Mr 70,000 heat shock genes in control or heat shock leukemic cells as correlated to their heat response. *Cancer Res* **50**: 2877–2884

Moan J (1990) On the diffusion length of singlet oxygen in cells and tissues. *J Photochem Photobiol B Biol* **6**: 343–347

Moan J, Berg K, Kvam E, Western A, Malik Z, Rück A and Schneckenburger H (1989) Intracellular localization of photosensitizers. In *Ciba Foundation Symposium 146*, Wiley, Chichester, pp 95–111

Mussarat J and Wani AA (1994) Quantitative immunoanalysis of pro-mutagenic 8-hydroxy-2′-deoxyguanosine in oxidized DNA. *Carcinogenesis* **15**: 2037–2043

Nordén B and Tjerneld F (1982) Structure of methylene blue-DNA complexes studied by linear and circular dichroism spectroscopy. *Biopolymers* **21**: 1713–1734

Olive PL and Banáth JP (1993) Induction and rejoining of radiation induced DNA single-strand breaks: tail moment as a function of position in the cell cycle. *Mutat Res (DNA repair)* **294**: 275–283

Olive PL, Banáth JP and Durand RE (1990) Heterogeneity in radiation-induced DNA damage and repair in tumour and normal cells measured using the comet assay. *Radiat Res* **122**: 86–94

Penning LC, Tijssen K, Boegheim JPJ, Vansteveninck J and Dubbelman TMAR (1994*a*) Relationship between photodynamically induced damage to various cellular-parameters and loss of clonogenicity in different cell-types with hematoporphyrin derivative as sensitizer. *Biochim Biophys Acta* **1221**: 250–258

Penning LC, Lagerberg JWM, Vandierendonck JH, Cornelisse CJ, Dubbelman TMAR and Vansteveninck J (1994*b*) The role of DNA damage and inhibition of poly(ADP-ribosyl)ation in loss of clonogenicity of murine L929 fibroblasts, caused by photodynamically induced oxidative stress. *Cancer Res* **54**: 5561–5567

Powell SN and McMillan TJ (1994) The repair fidelity of restriction enzyme induced double strand breaks in plasmid DNA correlates with radioresistance in human tumour cell lines. *Int J Radiat Oncol Biol Phys* **29**: 1035–1040

Roti Roti JL and Laszlo A (1988) The effects of hyperthermia on cellular macromolecules. In *Hyperthermia and Oncology*, Vol. 1, Urano M. (ed.), pp. 13–56. VSP: Zeist, The Netherlands

Tuite E and Kelly JM (1995) The interaction of methylene blue, azure B and thionine with DNA – formation of complexes with polynucleotides and mononucleotides as model systems. *Biopolymers* **35**: 419–433

Wagner SJ, Cifone MA, Murli H, Dodd RY and Myhr B (1995) Mammalian genotoxicity assessment of methylene blue in plasma – implications for virus inactivation. *Transfusion* **35**: 407–413

Warters RL and Stone OL (1983) Macromolecule synthesis in HeLa cells after thermal shock. *Radiat Res* **96**: 646–652

Ward JF (1988) DNA damage produced by ionising radiation damage in mammalian cells: identities, mechanisms of formation and repairability. *Progr Nuclear Acid Res Molec Biol* **35**: 95–125

Ward JF (1991) DNA damage and repair. *Basic Life Sci* **58**: 403–415

Welch WJ and Suhan JP (1985) Morphological study of the mammalian stress response: characterization of changes in cytoplasmic organelles, cytoskeleton and nucleoli and appearance of intra-nuclear actin filaments. *J Cell Biol* **101**: 1198–1211

West CML (1989) Size-dependent resistance of human spheroids to photodynamic treatment. *Br J Cancer* **59**: 510–514

Whitehurst C and Moore JV (1995) Development of an alternative light source for biomedical applications. *SPIE Proc* **2629**: 291–298

Wlodek D and Olive PL (1992) Neutral filter elution detects differences in chromatin organization which can influence cellular radiosensitivity. *Radiat Res* **132**: 242–247

© Cancer Research Campaign 1997